# Role of callose accumulation in the suppression of calcium-deficiency-induced necrosis in *Arabidopsis thaliana* cotyledons

**DOI:** 10.1080/15592324.2025.2607237

**Published:** 2026-01-06

**Authors:** Yusuke Shikanai, Takehiro Kamiya, Akihiro Saito, Kyoko Higuchi, Toru Fujiwara

**Affiliations:** a Department of Agricultural Chemistry, Tokyo University of Agriculture, Tokyo, Japan; b Department of Applied Biological Chemistry, Graduate School of Agricultural and Life Sciences, The University of Tokyo, Tokyo, Japan

**Keywords:** Calcium deficiency, necrosis, callose, cell death, Arabidopsis

## Abstract

Calcium (Ca) deficiency symptoms, such as blossom end rot in tomato and tip burn in lettuce, are among the most serious physiological disorders in agriculture. A common feature of this disorder is the expansion of necrosis. However, mechanisms underlying Ca-deficiency-induced necrosis remain poorly understood. We previously identified callose synthase genes (*GSL1*, *GSL8*, *GSL10*) as the causal genes of low-Ca-sensitive *Arabidopsis thaliana* mutants, which exhibit severe cell death in true leaves and reduced callose accumulation in cotyledons under low-Ca conditions. This raises the question of whether callose accumulation suppresses the spread of cell death. To clarify their relationship within the same organ, we examined callose deposition and cell death in the cotyledons of the *gsl10* mutant. Although the *gsl10* mutant showed a comparable level of total cell death to wild-type plants, the necrotic spots were larger. Furthermore, the largest necrotic spots were typically found at the cotyledon tip, but this tendency was weaker in *gsl10* mutant. Collectively, our results suggest that callose does not suppress the initiation of cell death but rather limits its propagation, thereby leading to the formation of a characteristic necrotic pattern preferentially occurring at the cotyledon tip.

## Introduction

Calcium (Ca) deficiency in crops represents one of the most serious physiological disorders in agriculture.^1^ Typical examples include blossom-end rot (BER) in tomato and tip burn in lettuce, which restrict cultivation practices and reduce yield. A characteristic of Ca deficiency disorders is the expansion of necrosis. In tomato BER, the distal portion of the fruit becomes water-soaked and turns black, whereas in tip burn, the leaf margins appear brown.

Several studies have addressed the mechanisms underlying necrosis under Ca deficiency. In tomato BER, analyzes of pectin methylesterases- or Ca^2+^/H^+^ exchanger-modified lines have proposed that a reduction in apoplastic soluble Ca leads to plasma membrane breakage and causes cell death.^2^^,^^3^ Scavenging reactive oxygen species by antioxidants such as glutathione and ascorbic acid has also been reported to contribute to BER suppression.^4^^,^^5^ In moss, mutant analyzes revealed that intermediate metabolites in the lignin biosynthetic pathway reduce tip burn-like symptoms.^6^ In Chinese cabbage, the Ca-binding protein calreticulin in the endoplasmic reticulum and the Ca channel CNGC have been involved in tip burn development.^7^^,^^8^ While many studies have focused on the cause of Ca deficiency-induced cell death, few studies have focused on mechanisms by which plants suppress the expansion of necrosis.

Callose, a *β*-1,3-glucan component of the cell wall, plays a key role in plant defense response against pathogen and wounding.^9^^,^^10^ However, to our knowledge, its role in suppressing necrosis has not been mentioned so far. We previously reported that *Arabidopsis thaliana* mutants defective in *glucan synthase-like* (callose synthase) genes exhibit severe necrosis under low-Ca conditions^11-13^ with transcriptional upregulation of defense response genes compared to the wild-type plants. In wild-type plants, ectopic callose deposition occurs under Ca deficiency in cotyledons and true leaves. In the mutants, reduced callose accumulation under low Ca has been most clearly observed in cotyledons, although necrosis has been primarily assessed in true leaves. Thus, there is a gap between the organ of characterization of necrosis (true leaves) and reduced callose (cotyledons) in our previous analyzes. It remained unclear whether callose induced by Ca deficiency suppresses the initiation of cell death, the subsequent expansion of necrosis, or both.

Here, we aimed to directly assess the relationship between callose deposition and necrosis development by monitoring both phenotypes in cotyledons. The *gsl10-5* mutant, which accumulates less callose under Ca deficiency, exhibited a total area of dead cells in cotyledons comparable to that of wild-type plants.^12^ However, the area of the largest necrotic lesion was significantly greater in *gsl10-5* cotyledons than in wild-type plants. This suggests that callose accumulation under low Ca does not influence the probability of individual cell death but instead functions to suppress the spread of necrosis to neighboring cells. Moreover, cell death in cotyledons preferentially occurred at the tip regions, and this spatial pattern was weaker in *gsl10-5* relative to wild-type plants. Taken together, these findings suggest that callose accumulation suppresses the expansion of necrosis and contributes to the spatial patterning of necrosis within cotyledons.

## Materials and methods

### Plant growth condition

*Arabidopsis thaliana* used in the study is from our lab stocks. *A. thaliana* seeds were surface-sterilized by commercial bleach and then sowed on agar plate containing Yamagami media^14^ with 1% (w/v) sucrose (84097-250G; Sigma-Aldrich Corp., St. Louis, MO, U.S.A.) and 1.5% (w/v) or 0.7% (w/v) purified agar (01056-15; Nacalai Tesque, Kyoto, Japan). Ca concentrations in media were adjusted by CaCl2. After sowing, agar plates were kept at 4 °C in the dark for 2 or more days. Plants were grown under 22 °C and 16 h light–8 h dark periods in a phytotron.

### Aniline blue staining and callose quantification

Aniline blue staining was performed as described previously.^12^ Briefly, shoots of *A. thaliana* seedlings grown for indicated days in indicated Ca concentrations were excised and immersed in approximately 200 μL of the fixative solution (methanol: pure water: acetic acid = 5:4:1 v/v/v;^15^ and incubated in 24 well plate in 4 °C for at least overnight. After removal of the fixative solution, about 400 μL 80 v/v % ethanol was added and incubated using heat block at 80 °C for about 10 min until the samples were decolorized. Then, after removal of ethanol, approximately 200 μL of chloral hydrate (chloral hydrate:pure water = 5:2 w/w) was added. The samples were incubated at 27 °C for overnight. After removal of the chloral hydrate solution, the samples were incubated with 1 M glycine (pH 9.5) at 4 °C for at least 3 h. The samples were then incubated with 0.1 mg/ml aniline blue (016-21302; Wako Pure Chemical Industries, Ltd.) in 1 M glycine (pH 9.5) for 2 h. Finally, the samples were mounted on glass slides with a 1:1 mixture of glycerol and 1 M glycine (pH 9.5) and then photographed using a Fluoview 1000 confocal laser scanning microscope(CLSM) (Olympus, Tokyo, Japan).

In CLSM observation, square images were acquired; each image was centered on the target cotyledon at an optical depth that showed the vascular bundles. The excitation wavelength was 405 nm, and emission wavelengths were from 480 to 530 nm. Each image was quantified using Fiji software; ^16^ the number of particles above the threshold level in the center of each image (i.e. the center square of a 3 × 3 grid) was counted using the “analyze particles” function. The threshold was set to ignore autofluorescence from dead cells, which was assessed using the samples that were treated in the absence of aniline blue. The number of particles was equivalent to the number of callose spots. The number of callose spots per 1-mm^2^ leaf area was quantified for subsequent analyzes. For 0.2 and 0.1 mM Ca, images with the median values of count of callose spots in each condition were presented as representative images. For 2 mM Ca, representative images were selected manually.

Experiments were repeated twice and both results were presented in this study. Statistical analyzes were performed against combined results of first and second experiments with ANOVA (objective variable: callose spot number per area, explanatory variable: day, genotype, and their interaction) using R.^17^

### Trypan blue staining and cell death analysis (Total dead area, largest necrotic spot area)

Trypan blue staining were performed as described previously.^12^^,^^18^^,^^19^ Briefly, shoots of *A. thaliana* seedlings grown for indicated days in indicated Ca concentrations were excised and immersed in 24-well plates filled with approximately 200  μL of lactophenol trypan blue solution (5 mL lactic acid, 5 g phenol, 5 mg trypan blue, 5 mL glycerol, 20 mL ethanol and 5 mL distilled water) and stained on a heating block at 90°C for 5–10 min. Next, the lactophenol trypan blue solution was removed from the wells and approximately 200 μL of chloral hydrate [chloral hydrate:distilled water = 5:2 (w/w)] was added to each well. For decolorization and clarification, the samples were incubated at 27 °C overnight. Samples were mounted on glass slides and photographed using fluorescence microscopy under bright-field mode. After photographing, each image was examined manually and cropped so as to one single image containing one single cotyledon for subsequent image analysis. Furthermore, images with bubbles were removed to avoid interference of image analysis. Images were rotated to the base of cotyledon (adjacent with petiole) locating the bottom of image so that the angle of each cotyledon can be identified as approximate ellipse subsequently.

Fiji software was used for quantification of images.^16^ “Color-threshold” function was used to identify the stained pixels and “Analyze Particle” was used to summarize the stained spots based on the thresholding by “Color threshold”. These functions were processed as macro (Process > Batch > Macro).

To identify the total dead area, color space (Hue, Saturation, Brightness) threshold was set to (120-200, 120-255, 0-190) and size threshold was set to over 20 pixels for removing the noise of staining. “Total area” in the output “Summary.csv” were used as the total dead area of each cotyledon.

To identify the largest necrotic spot, color space was the same as the case of total dead area, but the size threshold was set to 1000 pixels so as to ensure extracting spots with several neighboring cells dead (in this study, one typical cell showed about 100–200 pixels). After processed as macro, the output “Results.csv” was used to extract the largest particle as largest necrotic spot of each cotyledon. This “Results.csv” contains xy coordinates of the center of mass of each spot in original image, which subsequently used to identify the relative position from the center of mass of the approximate ellipse of cotyledons shape.

To identify the relative position of the largest necrotic spot from the center of mass of the approximate ellipse, each image was processed to select whole cotyledon area and run “analyze particle” with setting of “fit ellipse”. The center of mass, major axis (2 × major radius), minor axis (2 × minor radius), and the angle of major axis of approximate ellipse were obtained and used for the calculation of relative position of the largest necrotic spot.

Experiments were repeated twice and both results were presented in this study. Statistical analyzes were performed against combined results of first and second experiments with ANOVA using R.^17^ For 0.2 and 0.1 mM Ca, the representative image was selected as the sample showing the smallest root sum of squared deviations of the largest necrotic spot area and its x- and y-coordinates from their respective medians. For 2 mM Ca, representative images were selected manually.

### Measurement of Ca concentration

Col-0 plants were grown on agar plate containing Yamagami-medium with 2 or 0.2 mM Ca for 5 or 7 days, respectively. After indicated days, cotyledons were cut by razor blades on parafilm to avoid contamination of Ca from agar medium. The tip part and base part were collected, measured fresh weight and dry wight. Dried samples were digested by nitric acid (120 °C, 90 min) (cat. 28163-09, KANTO CHEMICAL co.,inc.). The residues were dissolved in 1 v/v% hydrochloric acid (cat. 18079-09, KANTO CHEMICAL co.,inc.) and used for atomic absorption spectrometry (AAS) (AA-6300; Shimadzu, Tokyo, Japan) in flame mode. As interference suppressant in Ca measurements in AAS, LaCl_3_ was supplemented in sample solution in the final concentration of 2000 ppm.

## Results

### Callose deposition time course in Arabidopsis cotyledons

**Figure 1.**
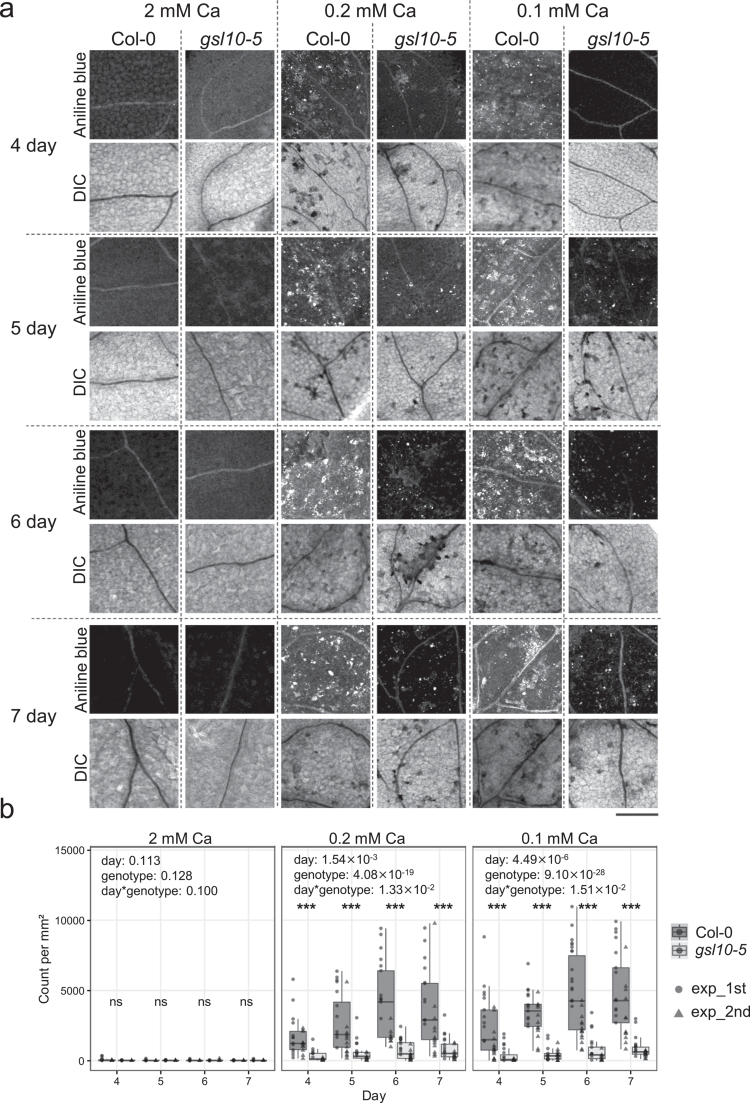
Callose accumulation in cotyledons of Col-0 and *gsl10-5.* (a) Representative images of aniline blue staining for callose visualization in cotyledons of Col-0 and *gsl10-5*. Plants were grown under 2, 0.2, 0.1 mM Ca for indicated days and then fixed and subjected to Aniline Blue staining. DIC, Differential interference contrast. Bar = 200 μm. (b) Callose spot of Col-0 and *gsl10-5* cotyledons under 2, 0.2, 0.1 mM Ca conditions. Plants were grown under indicated days and fixed, decolorized, clarified, and stained by aniline blue. The *p*-value from ANOVA (objective variable: callose spot number per area, explanatory variable: day, genotype, and their interaction) are shown in the top of each panel. Dark and light boxes indicate Col-0 and *gsl10-5,* respectively, and left (circle) and right (triangle) dots in each box indicate the results of first experiment and second experiment, respectively. *n* = 8~15 in each condition per experiment. Asterisks indicate significant differences between genotypes under each Ca condition and day (Student's *t*-test; ***P < 0.001). ns, not significant.

To investigate the relationship between necrosis development and callose accumulation in cotyledons, we obtained time-course data for both cell death and callose deposition using wild-type Col-0 and *gsl10-5* mutant, a weak allele of *GSL10* (AT3G07160) encoding callose synthase.^11^ In our previous study, ^11^ we demonstrated that the increased low-Ca-dependent necrosis in *gsl10-5* is caused by a mutation in *GSL10*. Two independent experiments were performed. To first confirm whether callose deposition is consistently reduced in *gsl10-5* mutant throughout the growth period examined, we performed aniline blue staining to visualize callose in cotyledons. Col-0 and *gsl10-5* were grown on medium containing 2, 0.2, or 0.1 mM Ca for 4–7 days. Cotyledons were imaged by confocal laser microscopy, and callose deposits were quantified as fluorescent puncta (Figure 1a, Supplemental Figure 1). Under 2 mM Ca, little callose accumulation was observed in either genotype (Figure 1a and b). In contrast, under 0.2 or 0.1 mM Ca, *gsl10-5* exhibited lower callose accumulation than Col-0 (Figure 1a and b). These results indicate that *gsl10-5* is defective in callose accumulation under Ca deficiency within the time frame of our experiments. Moreover, callose deposition increased progressively with time, indicating that prolonged Ca deficiency promotes enhanced callose accumulation.

### Callose deposition may suppress necrosis expansion but not the initiation of cell death

**Figure 2.**
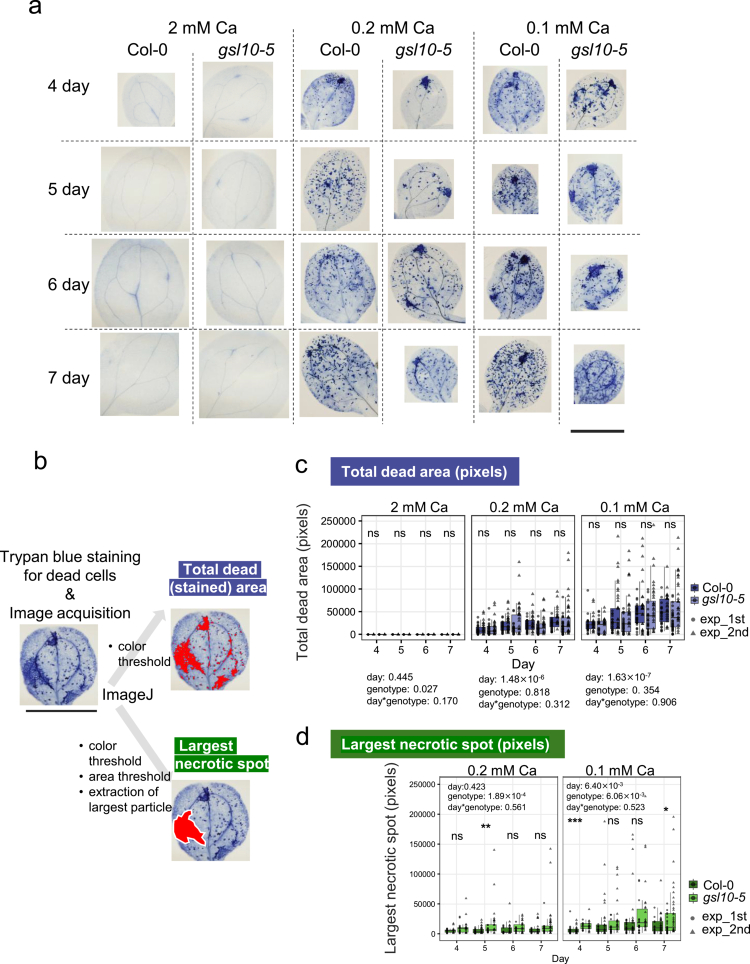
Largest cell death spot area in cotyledons of Col-0 and *gsl10-5.* (a) Representative images of trypan blue staining for cell death visualization in cotyledons of Col-0 and *gsl10-5*. Plants were grown under 2, 0.2, 0.1 mM Ca for indicated days and then stained with lacto-phenol trypan blue solution, then clarified and photographed. Bar = 1 mm. (b) Explanation of quantification methods of total dead area and largest necrotic spot in each cotyledon. Trypan blue staining images were photographed, then processed using ImageJ. For the measurement of largest necrotic spot, ImageJ function of “color threshold” and “analyze particle” was applied with the area threshold (over 1000 pixels). Then, from the output “Result.csv” file, the largest spot area in each cotyledon was identified. Bar = 1 mm. (c) Total dead area (pixels) of Col-0 and *gsl10-5* cotyledons under 2, 0.2, 0.1 mM Ca conditions. The *p*-value from ANOVA (objective variable: total dead cell area, explanatory variable: day, genotype, and their interaction) are shown above of each panel. Dark and light boxes indicate Col-0 and *gsl10-5,* respectively, and the left (circle) and right (triangle) dots in each box indicate the results of the first experiment and second experiment, respectively. (d) Largest necrotic spot (pixels). Plants were grown and stained in the same as (c). The *p*-value from ANOVA (objective variable: largest spot area, explanatory variable: day, genotype, and their interaction) are shown in the top of each panel. Dark and light boxes indicate Col-0 and *gsl10-5,* respectively, and left (circle) and right (triangle) dots in each box indicate the results of first experiment and second experiment, respectively. *n* = 6~27(2 mM), 17~39(0.1, 0.2 mM) in each condition per experiment. Asterisks indicate significant differences between genotypes under each Ca condition and day (Student's *t*-test; *P < 0.05; **P < 0.01; ***P < 0.001). ns, not significant.

We next asked whether callose accumulation suppresses the initiation of cell death or instead limits the subsequent expansion of necrosis. Cotyledons were stained with trypan blue to visualize dead cells (Figure 2a), and we quantified both the total area of cell death and the area of the largest necrotic lesion (Figure 2b). In this study, we defined “dead cell” as all cells stained by trypan blue and “necrosis” as 5-10 neighboring cells dead (see Materials and Methods). The total dead area was used as an indicator of the overall cell death, while the largest necrotic lesion represented necrosis expansion, a hallmark of Ca deficiency disorders.^6^^,^^20^^,^^21^ We found that the total cell death area did not differ significantly between Col-0 and *gsl10-5* (Figure 2c). By contrast, based on the results of the ANOVA and with several individual comparisons, the largest necrotic lesion tended to be larger in *gsl10-5* than in Col-0 (Figure 2d). These results are consistent with the idea that callose accumulation under Ca deficiency does not prevent the occurrence of cell death itself but contributes specifically to restricting the expansion of necrosis.

### Suppression of necrosis expansion by callose influences spatial patterning of necrosis

**Figure 3.**
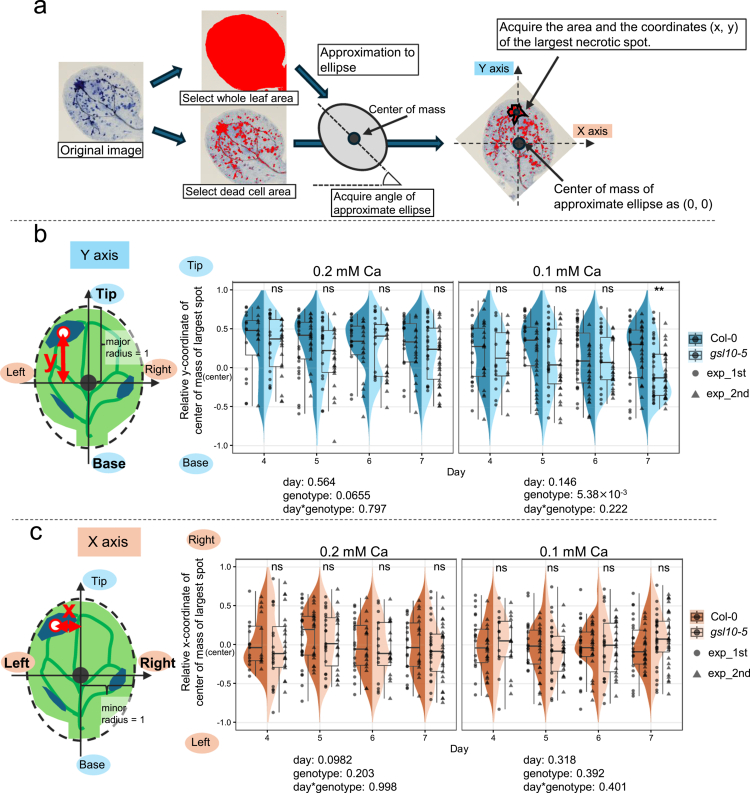
Analysis of position of the largest necrotic spot in cotyledons. (a) Explanation of methods of identification of xy coordinates of center of mass of largest necrotic spot. Trypan blue staining cotyledon images were processed in following two steps: (i) approximate cotyledon as ellipse, then acquire the angle of the ellipse, major radius, and minor radius. (ii) identify xy coordinate of the center of mass of largest necrotic spot (center of mass of the ellipse is used as origin (0, 0), tip and right are regarded as plus direction). (b) Y-coordinate of center of mass of the largest necrotic spot of Col-0 and *gsl10-5* cotyledons under 0.2, 0.1 mM Ca conditions. Length of major radius is regarded as 1. The *p*-value from ANOVA (objective variable: y-coordinate, explanatory variable: day, genotype, and their interaction) are shown above of each panel. Dark and light boxes indicate Col-0 and *gsl10-5,* respectively, and left (circle) and right (triangle) dots in each box indicate the results of first experiment and second experiment, respectively. (c) X-coordinate of center of mass of the largest necrotic spot. *n* = 18~37 in each condition per experiment. Asterisks indicate significant differences between genotypes under each Ca condition and day (Student's *t*-test; ** *P* < 0.01). ns, not significant.

During these observations, we noticed differences in the positional pattern of the largest necrotic lesions between genotypes. In Col-0, the large lesions were frequently located near the tip region of cotyledons, whereas in *gsl10-5*, they were often observed not only at tip but also in central regions (Figure 2a). To quantify this distribution, we reanalyzed the same dataset of images presented in Figure 2 and expressed the lesion position as relative coordinates along the short (x-axis) and long (y-axis) axes of an ellipse approximating the cotyledon shape (Figure 3a). Along the y-axis (Figure 3b), Col-0 exhibited a strong tendency for lesions to occur near the tip, whereas this tendency was reduced in the *gsl10-5*. Notably, under 0.1 mM Ca, *gsl10-5* displayed lesions predominantly in central regions rather than at the tip (Figure 3b). Along the x-axis, lesions in both genotypes were concentrated near the center (Figure 3c). These results suggest that the suppression of necrosis expansion by callose contributes to the spatial patterning of necrosis.

To test whether the preferential occurrence of necrosis at the cotyledon tips might be explained by the Ca distribution, we measured Ca concentrations in the basal and tip regions using atomic absorption spectrometry. Cotyledons of Col-0 were harvested at comparable sizes: after 5 days at 2 mM Ca and after 7 days at 0.2 mM Ca. The Ca concentration in the tip region was not reduced compared to that in the base region under either condition (Supplemental Figure 2).

## Discussion

In our previous analyzes of *A. thaliana* callose synthase mutants, callose deposition was primarily examined in cotyledons, whereas cell death was assessed in emerging leaves, leading to an organ-dependent discrepancy in interpretation. In this study, we examined the relationship between callose deposition and necrosis in cotyledons under Ca-deficient conditions.

Although the total area of cell death in *gsl10-5* cotyledons was not higher than that in Col-0, the larger necrotic lesion was larger in *gsl10-5*than in Col-0 (Figure 2). Considering that both the total area of cell death and callose deposition increased progressively with time, these results suggest that cell death may induce callose deposition, which, in turn, prevents the spread of cell death into surrounding tissue. A possible scenario is that Ca deficiency damages cell walls or plasma membranes, thereby generating damage-associated molecular pattern(s) (DAMP), such as oligogalacturonides (OGs) and PEPs. These DAMPs may trigger Ca^2+^ influx into the cytosol, causing defense responses, leading to callose deposition, while simultaneously promoting cell death. Indeed, OGs have been reported to accumulate in potato plants grown under Ca-deficient conditions.^22^ Previous studies have reported that Ca-deficient conditions elevate cytosolic Ca concentration, which is known as a signaling messenger for inducing defense responses, supporting this possible scenario.^23^^,^^24^ Further elucidation of the relationship between callose and cell death under Ca deficiency will likely require ultrastructural analyzes of callose deposition as well as examination of known DAMPs in relation to Ca deficiency disorders.

What would be the functional similarity or difference to other *GSL* genes in response to biotic and abiotic stress? There are 12 *GSL* genes in Arabidopsis genome. We previously demonstrated that *GSL1*, *GSL8*, and *GSL10* are essential and function redundantly to suppress necrosis under low-Ca conditions.^11-13^ Numerous studies have examined the relationship between *GSL* genes or callose itself and necrosis or cell death under pathogen infection. A representative example is *PMR4*/*GSL5*, the causal gene of the *pmr4* mutant, which shows resistance to powdery mildew.^9^^,^^10^^,^^25^ Although there is no direct evidence showing that callose synthesized by PMR4/GSL5 suppress necrosis itself, many studies have described its involvement in biotic stress tolerance. For example, overexpression of *GSL5* leads to strong resistance by enhancing callose deposition and thereby physically blocking fungal hyphal penetration.^26^ Because our growth conditions are sterile, leaving no opportunity for pathogen invasion, the mechanisms by which *GSL10* in suppression of necrosis under low-Ca conditions may differ from mechanisms by which *GSL5* suppresses pathogen invasion. Alternatively, a plasmodesmal callose, regulating intercellular transport, are also highly likely to be involved in the development of necrosis. GSL8 and GSL12, which mediate callose accumulation of plasmodesmata, have been shown to control the movement of proteins in Arabidopsis,^27^^,^^28^ clearly indicating the involvement of GSL proteins in regulating intracellular transport. Moreover, it has been demonstrated that plasmodesmal callose restricts viral movement and the size and number of lesion,^29^ indicating that intercellular movement of molecules is a well-established factor influencing the spread of necrosis. Taken together, these observations suggest that callose accumulation mediated by GSL10 may restrict the spread of necrosis through a mechanism analogous to its restriction of viral movement. Identifying the signaling molecules that promote necrosis propagation under low-Ca conditions remains an important future question. Possible candidates include endogenous DAMPs or certain proteins.

Tip burn, a Ca-deficiency disorder in leafy vegetables, manifests as necrosis at the distal margins of the young leaves. This phenomenon is generally attributed to the low mobility of Ca in the transpiration stream, resulting in insufficient delivery to rapidly expanding distal tissues. Here, by analyzing spatial patterns of cell death in *A. thaliana* cotyledons, we found that necrosis also tended to initiate at the tip regions of the wild-type plants, suggesting that the cotyledons of *A. thaliana* and young leaves of crop species may share similar mechanisms of necrosis development under Ca deficiency.

In our Ca concentration measurements, the tip and basal regions of the cotyledons did not show consistently lower Ca levels at the tips. This observation is consistent with previous studies on lettuce, which reported that leaves exhibiting tipburn do not necessarily have reduced Ca concentrations at the tip regions relative to basal tissue.^30^ In our observations, necrosis at the cotyledon tips frequently occurred at the intersections of vascular bundles, likely near the hydathodes. Considering that Ca transport primarily occurs through the xylem via the transpiration stream, the amount of soluble Ca available near hydathodes – before being sequestered into pectins – may be a critical determinant of the onset of cell death. A recent report showing that hydathodes capture Ca in xylem fluid before guttation supports this idea.^31^ The function of callose in suppressing necrosis, together with the preferential occurrence of necrosis in the tip region, may shape the spatial pattern of Ca-deficiency-induced necrosis in leaves.

## Supplementary Material

Supplementary material Suuplemental Figures

Supplementary material Supplemental figures
