# Supplementary material for: Role of callose accumulation in the suppression of calcium-deficiency-induced necrosis in Arabidopsis thaliana cotyledons
Source: Plant Signal Behav. 2026 Jan 6;21(1):2607237. doi: 10.1080/15592324.2025.2607237 (PMC12785192; doi:10.1080/15592324.2025.2607237)
Supplement: Supplementary material — Suuplemental Figures [file KPSB_A_2607237_SM7451.pdf]

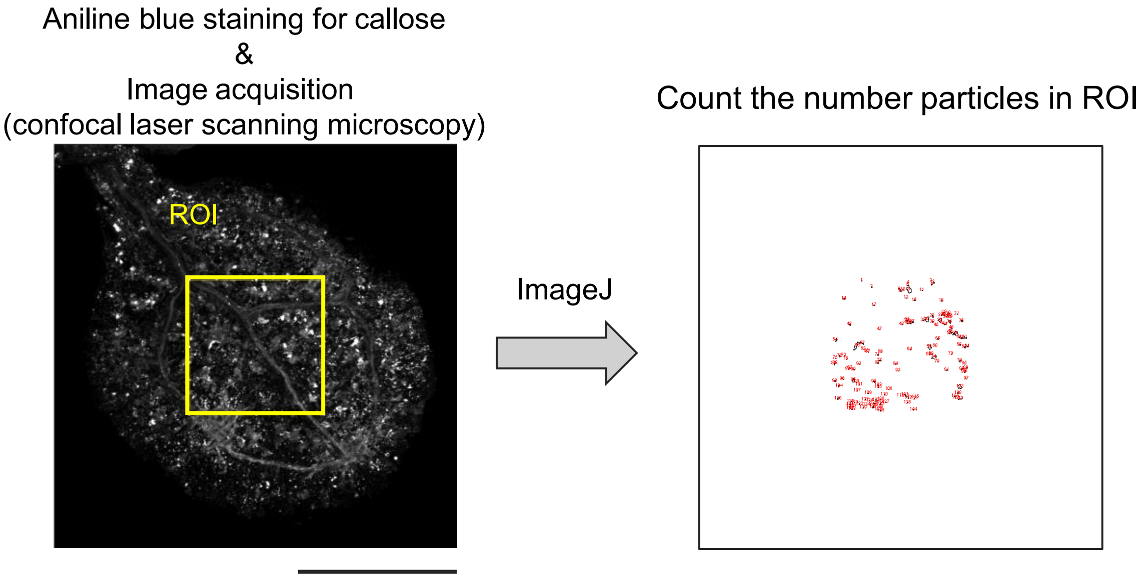

Supplemental Figure 1 Schematic diagram of image analysis of callose quantification

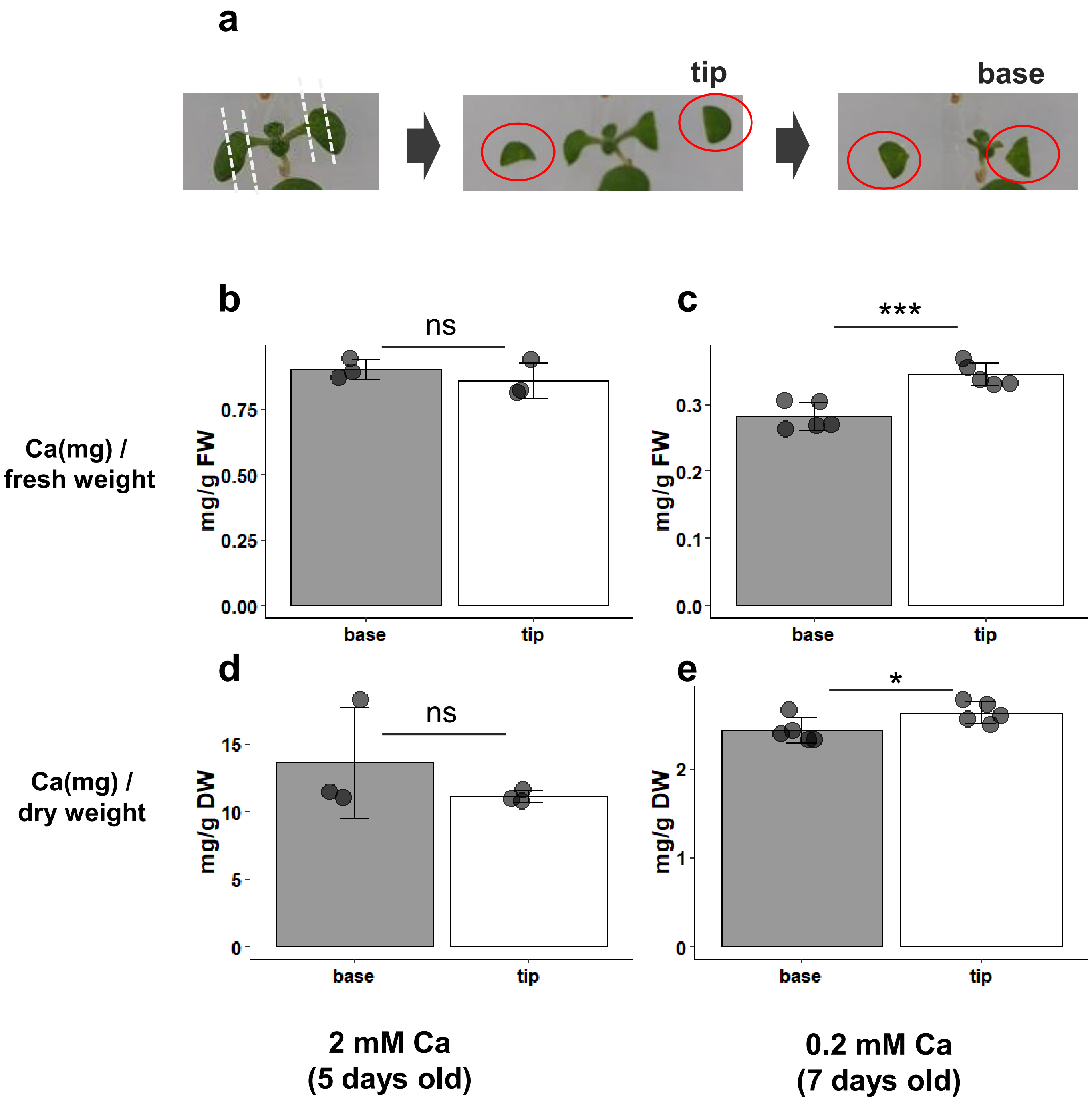

Supplemental Figure 2 Calcium concentration in the tip or base of cotyledons
