# Supplementary material for: Role of callose accumulation in the suppression of calcium-deficiency-induced necrosis in Arabidopsis thaliana cotyledons
Source: Plant Signal Behav. 2026 Jan 6;21(1):2607237. doi: 10.1080/15592324.2025.2607237 (PMC12785192; doi:10.1080/15592324.2025.2607237)
Supplement: Supplementary material — Supplemental figures [file KPSB_A_2607237_SM0843.docx]

**Supplemental figure 1 Schematic diagram of image analysis of callose quantification**

Explanation of quantification methods of callose. Aniline blue staining images were photographed using confocal laser scanning microscopy as the center square of 3 x 3 grids of images were covered in cotyledons. Then, using ImageJ function of “analyze particle”, the number of the particle above threshold were counted as the number of callose spot and represented as count per mm^2^. Bar = 500 μm.

**Supplemental figure 2 Ca concentration in the tip or base of cotyledons**

(a) Sampling of tip region and base region of cotyledons. (b-e) Ca content per fresh weight or dry weight of base or tip region in Col-0. Plants were grown under 2 mM Ca or 0.2 mM Ca for 5 or 7 days, respectively. After weighing fresh weight, samples were dried, weighed dry weight, digested by nitric acid, and measured the concentration of Ca using atomic absorption spectrometry. n=3 (2 mM Ca), 5 (0.2 mM Ca). Asterisk(s) indicate significant differences between base and tip (student-t test; * <0.05, ***<0.001, ns means no significant).
